# Home practice in mindfulness-based interventions for psychosis groups: a systematic review and qualitative study

**DOI:** 10.1186/s40359-021-00694-4

**Published:** 2022-01-12

**Authors:** Pamela Jacobsen, Twinkle Choksi, Katherine Sawyer, Cassia Maximen, Emma Harding, Matthew Richardson

**Affiliations:** 1 Department of Psychology, University of Bath, Bath, BA2 7AY UK; 2 South London and Maudsley NHS Foundation Trust, London, UK

**Keywords:** Mindfulness, Psychotic disorders, Qualitative research, Treatment outcomes, Systematic review

## Abstract

**Background:**

Regular home practice is considered a core component of mindfulness groups and may be associated with better treatment outcomes. This study aimed to (1) review the existing evidence on how much home practice people do in mindfulness-based interventions for psychosis groups, and (2) explore participants’ experiences of the barriers and facilitators to completing home practice in a mindfulness for psychosis group using a qualitative study.

**Methods:**

In study 1, we conducted a systematic review of mindfulness-based interventions for psychosis studies and extracted data on home practice rates. In study 2, we conducted semi-structured interviews with people who had completed a mindfulness for psychosis group (N = 5) as part of their routine community care, specifically focusing on experiences of home practice.

**Results:**

Out of 43 studies included in the systematic review, only 5 reported any data on amount of home practice, and none examined the relationship between completion of home practice and treatment outcomes. In the qualitative study, participants described home practice as being difficult but important. Arising themes were similar to findings from previous (non-psychosis) studies suggesting that generic challenges are common, rather than being specific to psychosis.

**Conclusions:**

We recommend that future mindfulness-based interventions for psychosis studies record data on home practice rates, in order to investigate any association between home practice and treatment outcome. Our qualitative findings suggest home practice can be a valued part of mindfulness for psychosis group, and a normalising approach could be taken when and if participants encounter common challenges.

**Supplementary Information:**

The online version contains supplementary material available at 10.1186/s40359-021-00694-4.

## Background

Mindfulness-based interventions for psychosis have been shown to have many benefits according to recent meta-analyses, including reducing psychotic and affective symptoms, and reducing risk of hospitalisation [1, 2]. Like other mindfulness-based interventions, it is typically delivered in a group format, which is experiential in nature, with each session including in-group mindfulness practice followed by teacher-led enquiry. Mindfulness-based interventions for psychosis also includes adaptations to meet the particular needs of people with psychotic experiences, such as reduced length of meditation practices (usually around 10 min), and more frequent guidance during practices including explicit reference to working with experiences such as voices and paranoia [3]. Another adaptation is a reduced emphasis on home practice, out of recognition that people with psychosis might face additional barriers to practising without the support of a group, and the mindfulness therapist present [4]. This may be due to the particularly distressing nature of their symptoms (e.g. hearing critical and hostile voices [5]), as well as cognitive difficulties such as reduced concentration levels [6].

However, it is not yet known whether a decreased emphasis on home practice might actually be stopping participants from getting the most out of a mindfulness for psychosis group given that mindfulness is conceptualised as a skill which is best acquired with frequent practice [7, 8]. For example, in Mindfulness-Based Stress Reduction (MBSR; [7]) and Mindfulness-Based Cognitive Therapy (MBCT; [8]) the curriculum includes home practice requirements of around 45 min a day, 6 days a week for the duration of the 8-week group. There is also an important distinction made between ‘formal’ (e.g. body scan) and ‘informal’ (e.g. mindfulness of everyday activities) home practice in these programmes. Formal mindfulness practises are seen as vital in helping participants to develop key skills. These include how to deliberately turn towards moment-by-moment experience, bring awareness to habitual patterns of responding, and make skilful choices when deciding how to respond, in order to step out of ‘automatic pilot’ mode. This formal practice lays the foundation for these skills to be deployed when needed in everyday situations, such as when encountering a stressful event. Kabat-Zinn uses the metaphor of regular formal practice as ‘weaving your parachute everyday’, so it is ready when you need it [7]. This is supported by empirical data from a study by which found that on days when people completed a formal meditation home practice, they responded with greater mindfulness to daily events, which in turn was associated with better psychological well-being [9].

There is evidence from the MBCT for depression literature that increased completion of weekly home practice is associated with better treatment outcome. Crane et al. [10] analysed home practice diaries for 99 participants in a MBCT trial and found that participants who practised on at least three days a week were almost half as likely to have relapsed at 12-month follow-up, compared to people practising < 3 days a week. A subsequent meta-analysis of 28 MBCT/MBSR studies in a range of both clinical and non-clinical samples, also reported a statistically significant association between increased home practice and better treatment outcomes (r = 0.26, 95% CI 0.19–0.34) [11]. Lloyd et al. [12] further conducted a systematic review into controlled trials of MBCT or MBSR groups, restricting their search to only studies which recorded and reported data on home practice completion. They found seven trials with available data on the association between home practice completion and treatment outcome, with four of those reporting a positive association, and three showing no effect.

Despite some equivocal findings, which are to be expected in a heterogeneous literature, the evidence overall supports the premise that formal home practice is an important part of Mindfulness-Based Interventions. Participants should therefore be encouraged to complete the home practice as set in order to get maximum benefit. However, given the time demands and challenging nature of mindfulness practice, it is perhaps no surprise that participants often struggle to complete home practice. For example, Parsons et al. [11] reported an average of 64% of home practice completed as set in their meta-analysis of 43 studies. An online survey of people who practised mindfulness regularly (N = 218), found that common barriers to home practice including encountering challenges in the practice (e.g. falling asleep), and feeling reluctant to practice when aversive experiences such as boredom or irritation were present [13]. Despite the importance of home practice, meta-syntheses of multiple in-depth qualitative studies show that participants’ experiences of what helped or hindered them in home practice has not yet been investigated in any detail [14, 15]. More data is therefore needed on how to best support people to successfully meet commonly encountered challenges in completing home practice.

In summary, we do not yet know whether home practice completion is associated with better treatment outcome in psychosis, as has been found in depression, and how advice and support with completing home practice can best be tailored for this particular clinical group.

The aims of the 2 sequential studies reported in this paper were therefore:

1. To conduct a systematic review on what is currently known about home practice in mindfulness-based interventions for psychosis groups and any association with treatment outcome
2. To conduct a qualitative study of people’s experience of the facilitators and barriers to doing home practice during a mindfulness for psychosis group.

## Study 1: Systematic review of home practice in mindfulness-based interventions for psychosis groups

### Method

#### Review questions

1. (Primary—quantitative) How much formal home practice do people report doing during participation of a mindfulness for psychosis group?
2. (Secondary—qualitative) What are the reported facilitators/barriers to formal home practice for people taking part in a mindfulness for psychosis group?

A review protocol was written and pre-registered on the Open Science Framework before the searches were run (https://doi.org/10.17605/osf.io/cgakp; 12th March, 2019).

#### Searches

We initially conducted searches for mindfulness-based interventions for psychosis studies in the electronic databases Scopus and PubMed for peer-reviewed journal articles published in English up to 31st December 2018. Additional search terms were added to address the secondary review question, as we anticipated that some relevant data might be published as separate qualitative or mixed-methods papers (see Additional file 1 for complete list of search terms). Initial searches were run between March and June 2019. The systematic review was later updated, with searches being run up to 12th October 2021 (run in October 2021).

#### Inclusion/exclusion criteria

For the primary review question any study design was included, so long as empirical data was reported (i.e. excluding commentary or review papers). This included randomized controlled trials, non-randomized controlled trials, and uncontrolled studies. Likewise, for the secondary review question, any study design was included, so long as empirical data was reported, including mixed methods studies and solely qualitative studies. Studies were included if participants were at least 16 years old and were taking part in mindfulness-based interventions for psychosis groups, regardless of diagnosis or symptom profile. Studies were eligible for inclusion if they described and/or evaluated a mindfulness group intervention (of however many sessions or duration of treatment), including at least one formal guided practice per group session with teacher-led inquiry after each practice. Therapies described predominantly as compassion- or acceptance based were not included if they did not meet these criteria.

#### Study selection and data extraction

**Fig. 1:**
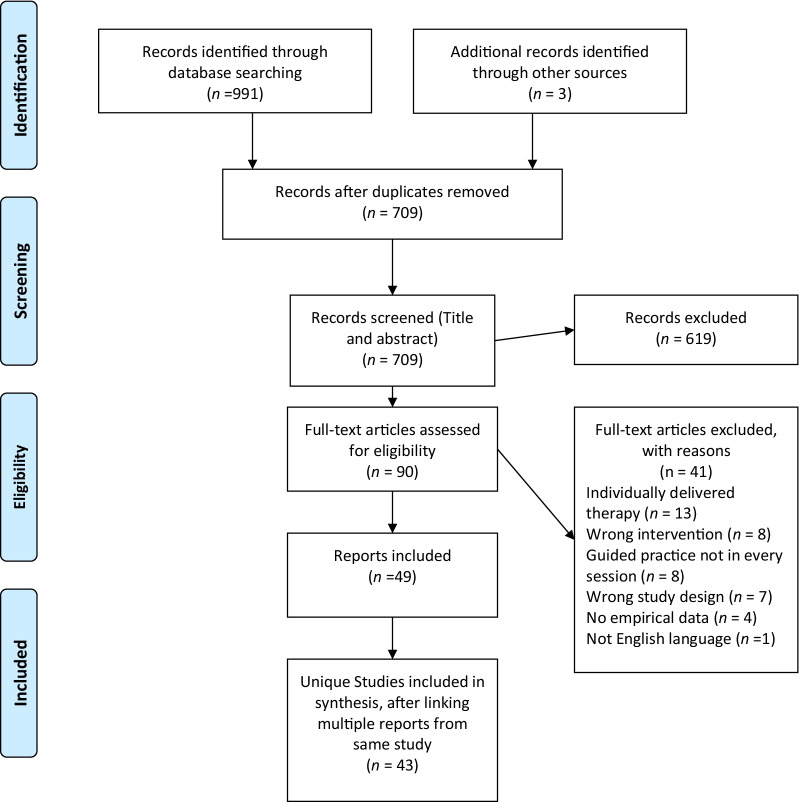
PRISMA flow chart of primary search

One reviewer first screened all titles and abstracts identified from searches to determine eligibility. At the full-text screening stage, two reviewers independently screened articles for inclusion, with any discrepancies resolved by discussion. Where needed, corresponding authors were contacted to ask for any missing data that could have helped to assess eligibility. Reasons for exclusion at the full-text screening stage were recorded. For included studies, multiple reports from the same study, based on the same underlying data from the same participants, were linked together. We linked studies based on matching trial registration numbers where available, references to linked papers in the reports themselves, and consultation with authors where required. For summary of searches see PRISMA diagrams: Fig. 1 (primary review question) and Fig. 3 (secondary review question; Additional file 1). We used a standardised data extraction template to record relevant information from each included study, with a sample double-checked for consistency.

#### Quality assessment

The quality of eligible studies was assessed using the Mixed Methods Appraisal Tool (MMAT; [16]). The MMAT is designed to assess quantitative, qualitative and mixed methods studies using a single integrated tool which also incorporates criteria for assessing RCTs in line with the Cochrane criteria [17]. A summary score is calculated by dividing the number of criteria definitely met (i.e. scored as a ‘yes’) divided by 4 and expressed as a percentage. Quality scores therefore ranged from 0 to 100%.

#### Data synthesis

For the primary quantitative question, we planned to calculate the range and mean average of the proportion of participants who reported doing any formal home practice, and the pooled average of the numbers of minutes per day, or average numbers of sessions of practice per week, over the course of the group. For the secondary qualitative question, we planned to report a narrative synthesis of qualitative data reporting facilitators and barriers to formal home practice.

## Results

### Primary review question: how much formal home practice do people report doing during participation of a mindfulness for psychosis group group? (Quantitative)

#### Study characteristics

Table 1 show the characteristics of studies included in the primary review question (*n* = 43). A range of study designs were included: Randomised controlled trials (RCTs; *n* = 18), non-randomised controlled trial (*n* = 3), uncontrolled pre-post studies (*n* = 19), qualitative studies (*n* = 3). In studies that used a comparison group (*n* = 21) these were: Treatment as Usual (TAU, n = 14), waitlist (n = 2), active control (n = 3) and both active control + TAU comparison groups (n = 2). Quality assessment using the Mixed Methods Appraisal Tool (MMAT) resulted in 4/43 studies being assessed as low quality (scoring 0% or 25%), 14/43 as medium quality (scoring 50%) and 25/43 as high quality (scoring 75% or 100%).

**Table 1:** Characteristics of studies included for primary review question (n = 43)

References Design Trial Registration Number Country	Setting	Treatment group and sample size	Comparison group and sample size	Treatment time	Primary outcomes	MMAT Section assessed under	MMAT score (%)
1. Böge et al. [28] Qualitative Germany	Inpatient	Mindfulness Based Group Therapy, *n* = 27	None	3 × 30-60 min session/week for 4 weeks	Participant experiences	1. Qualitative	100
2. Böge et al. [59] RCT NCT03671005 Germany	Inpatient	Mindfulness Based Group Therapy, *n* = 21	TAU, *n* = 19	3 × 30-60 min session/week for 4 weeks	Feasibility and acceptability	2. RCT	100
3. Brown et al. [18] Qualitative USA	Outpatient rehabilitation centre	Mindfulness Based Stress Reduction, *n* = 15	None	2 × 60 min session/week for 8 weeks	Participant experiences	1. Qualitative	50
4. Çetin and Aylaz [60] Non- Randomised controlled trial Turkey	Community mental health services	Mindfulness based Psychoeducation, *n* = 55	TAU, *n* = 80	2 × 70 min sessions/week for 4 weeks	Insight (BCIS)	3. Quantitative Non-Randomised	50
5. Chadwick et al. [4] Pre-post study UK	Community mental health services	Mindfulness Groups for Psychosis, *n* = 11	None	1 × 90 min session/week for 6 weeks	General psychological distress (CORE)	4. Quantitative Descriptive	100
6. Chadwick et al. [61] RCT UK	Community mental health services	Mindfulness Groups for Psychosis, *n* = 11	Waitlist, *n* = 11	1 × 90 min session/week for 5 weeks (followed by 5 weeks home practice)	General psychological distress (CORE)	2. RCT	0
7. Chadwick et al. [62] RCT ISRCTN74054823 UK	Community mental health services	Group Person Based Cognitive Therapy, *n* = 54	TAU, *n* = 54	1 × 90 min sessions/week for 12 weeks	General psychological distress (CORE)	2. RCT	100
8. Chien and Lee [63] RCT Hong Kong	Outpatient clinics	Mindfulness based Psychoeducation group program, *n* = 48	TAU, *n* = 48	1 × 120 min session/2 weeks for 12 weeks	Symptom severity (BPRS)	2. RCT	50
9. (a) Chien and Thompson [64] (b) Wang et al. [65] (c) Chien et al. [66] (d) Chien et al. [67] (e) Chien et al. [68] RCT NCT01667601 Hong Kong, Taiwan and China	Outpatient clinics	Mindfulness based Psychoeducation group program, *n* = 114	Conventional Psychoeducation group programme, *n* = 114, TAU *n* = 114	1 × 120 min session/2 weeks for 24 weeks	Re-hospitalisation and psychotic symptoms (PANSS)	2. RCT	50
10. Dannahy et al. [69] Pre-post study UK	Not stated	Person based cognitive therapy, *n* = 62	None	1 × 90 min session/week for 8–12 weeks	General psychological distress (CORE)	4. Quantitative Descriptive	100
11. Davis et al. [19] Qualitative USA	Community mental health services	Mindfulness for anxiety, *n* = 5	None	2 × 60 min session/week for 8 weeks	Participant experiences	1. Qualitative	25
12. Davis et al. [20] RCT USA	Outpatient rehabilitation centre	Mindfulness intervention for rehabilitation and recovery in Schizophrenia, *n* = 18	Intensive support, *n* = 16	2 × 75 min sessions/week for 8 weeks, completed twice	Feasibility and acceptability	2.RCT	75
13. Ellett et al. [70] RCT UK	Not stated	Mindfulness for psychosis, *n* = 14	TAU, *n* = 13	12 sessions (duration and frequency not stated)	Feasibility and acceptability	2. RCT	50
14. (a) Hickey et al. [71] (b) Hickey et al. [72]	Community mental health services	Mindfulness and compassion program	None	1 × 90 min sessions/week for 8 weeks	Feasibility and acceptability	4. Quantitative Descriptive	100
15. Jacobsen et al. [23] Pre-post study UK	Specialist psychosis inpatient unit	Mindfulness groups for psychosis, *n* = 8	None	1 × 60 min class/week for 6 weeks	Feasibility and acceptability	4. Quantitative Descriptive	75
16. Jacobsen et al. [40] Pre-post study UK	Community mental health services	Mindfulness groups for psychosis, *n* = 34	None	1 × 90 min sessions/week for 8 weeks	Stress and symptom related distress (self- report visual analogue scale)	4. Quantitative Descriptive	100
17. Johnson et al. [21] Pre-post study USA	Outpatients	Loving Kindness Meditation, *n* = 18	None	1 × 60 min session/week for 6 weeks, plus one booster session 6 weeks later	Feasibility and acceptability	4. Quantitative Descriptive	100
18. Jones et al. [73] Pre-post study UK	Specialist secondary mental health care service	Person-Based Cognitive Therapy, n = 95	None	1 × 90 min/week for 12 weeks	Engagement, outcomes, service costs	4. Quantitative Descriptive	100
19. Khoury et al. [38] Pre-post study Canada	Early intervention service	Compassion, acceptance and mindfulness, n = 17	None	1 × 60-75 min/week for 8 weeks	Feasibility and acceptability	4. Quantitative Descriptive 5. Mixed Methods	100 100
20. Lam et al. [22] RCT NCT03632278 Hong Kong	Outpatient clinics and residential care setting	Mindfulness Based Stress Reduction, *n* = 26	TAU, *n* = 26	1 × 90 min session/week for 8 weeks	Emotion regulation (ERQ and SRRS)	2. RCT	100
21. Langer et al. [74] RCT Spain	Not stated	Mindfulness Based Cognitive Therapy, *n* = 11	Waitlist, *n* = 12	1 × 60 min session/week for 8 weeks	Feasibility and psychotic symptoms (CGI-SCH)	2. RCT	25
22. Langer et al. [75] RCT ISRCTN24327446 Chile	Clinical centres	Mindfulness Based Cognitive Therapy, *n* = 24	TAU, *n* = 21	1 × 90 min session/week for 8 weeks	Cognitive functioning	2. RCT	0
23. Lee [76] RCT Taiwan	Rehabilitations wards and daycare centres in psychiatric hospitals	Mindfulness Based Intervention, *n* = 30	TAU, *n* = 30	1 × 90 min/week for 8 weeks	Psychotic symptoms (PANSS)	2. RCT	50
24. López-Navarro et al. [77] RCT Spain	Community rehabilitation centre	Integrated rehabilitation treatment and mindfulness-based intervention, *n* = 22	Integrated rehabilitation treatment, *n* = 22	1 × 60 min sessions/week for 26 weeks	Health related psychological quality of life (WHOQOL-BREF)	2. RCT	50
25. (a) López-Navarro et al. [78] (b) López-Navarro et al. [79] RCT ISRCTN52873519 Spain	Community rehabilitation centre	Integrated rehabilitation treatment and mindfulness-based intervention, *n* = 26	Integrated rehabilitation treatment, *n* = 26	1 × 60 min sessions/week for 26 weeks	Inhibitory control (SCWT) Psychotic symptoms (PANSS)	2. RCT	50
26. MacDougall et al. [80] RCT NCT02342210 Canada	Outpatients in prevention and early intervention programme	Mindfulness Ambassador programme, *n* = 11	TAU, *n* = 10	1 × 60 min session/week for 12 weeks	Feasibility and acceptability	2. RCT	100
27. Martins et al. [81] Pre-post study Portugal	Community mental health services	Compassion, mindfulness and accepting approach to psychosis, *n* = 7	None	1 × 60 min session/week for 5 weeks	Feasibility and acceptability	4. Quantitative Descriptive	50
28. Mediavilla et al. [82] Pre-post study NCT03434405 Spain	Outpatients	SocialMind, *n* = 25	None	1 × 90 min session/week for 8 weeks	Feasibility and acceptability	4. Quantitative Descriptive	100
29. Millar et al. [37] Pre-post study UK	Inpatient (rehabilitation wards)	Mindfulness group*, n* = 35	None	3 × 30 min session/week (open-ended)	Acceptability and participant experience	4. Quantitative Descriptive	100
30. Moorhead [24] Pre-post study UK	Early intervention service	Mindfulness for psychosis, *n* = 19 (11 service users, 3 carers, 5 staff)	None	1 × 60 min session/week for 8 weeks	Acceptability and general psychological distress (CORE)	4. Quantitative Descriptive	100
31. Özdemir and Budak [83] RCT Turkey	Community mental health centres	Mindfulness Based Stress Reduction, *n* = 50	Psychoeducation, *n* = 50, TAU, *n* = 56	1 × 30 min session/week for 8 weeks	Hope (HHS)	2. RCT	50
32. Randal et al. [84] Pre-post study UK	Community mental health services	Mindfulness Based Cognitive Therapy for Psychosis, *n* = 21	None	1 × 120 min session/week for 8 weeks	Change in sense of self and experience of psychosis (repertory grids)	4.Quantitative Descriptive	75
33. Ryan et al. [85] Pre-post study Ireland	Psychiatric hospital (inpatient and outpatient)	Living Through Psychosis programme, n = 64	None	1 × 300 min, then 7 × 180 min sessions over 4 weeks	Feasibility, acceptability and emotion regulation (DERS)	4.Quantitative Descriptive	75
34. Samson and Mallindine [25] Pre-post study UK	Early intervention service	Mindfulness groups for psychosis, *n* = 10	None	1 × 90 min session/week for 8 weeks	Feasibility and acceptability, general psychological distress (CORE)	4.Quantitative Descriptive	100
35. Shen et al. [86] RCT ChiCTR2100043803 China	Inpatient services (rehabilitation ward)	Mindfulness Based Intervention, n = 50	TAU, n = 50	5 × 45 min session/week for 6 weeks	Psychotic symptoms (PANSS)	2. RCT	100
36. Sheng et al. [46] Pre-post study ChiCTR‐OOB‐17,014,038 China	Inpatient services	Mindfulness meditation, *n* = 5	None	1 × 90 min session/week for 8 weeks (with 8 monthly follow-up sessions)	Psychotic symptoms (PANSS)	4. Quantitative Descriptive	50
37. Spidel et al. [26] RCT USA + Canada	Community mental health services	Acceptance and Commitment Therapy + Mindfulness, *n* = 30	TAU, *n* = 20	1 × 90 min session/week for 8 weeks	Emotional regulation and psychotic symptoms (CERQ and BPRS-E)	2.RCT	50
38. Tang et al. [87] RCT China	Inpatient	Mindfulness Based Cognitive Therapy, *n* = 31	TAU, *n* = 31	1 × session (duration not stated)/week for 8 weeks	Self-stigma (Link’s stigma scales)	2. RCT	50
39. Ting et al. [36] Pre-post study Hong Kong	Outpatients	Mindfulness for Psychosis, *n* = 22	None	1 × 75 min session/week for 4 weeks	Feasibility and acceptability	4. Quantitative Descriptive	100
40. Tong et al. [27] Pre-post study NCT02244970 Hong Kong	Early intervention services	Mindfulness Based intervention, *n* = 14	None	1 × 90 min sessions/week for 7 weeks	Depression and anxiety symptoms (DASS-21)	4. Quantitative Descriptive	75
41. (a) Usher et al. [88] RCT NCT02398292 (b) Thompson et al. [29] Qual USA	Early intervention services	Meals, mindfulness, and Moving forward M^3^, n = 17 (*n* = 13 in qual study)	TAU, *n* = 16	1 × 30 min session (within longer 4 h session with varied activities)/week for 12 weeks	Attendance and participant experiences	3. Quantitative non-randomised 5. Qualitative	75 100
42. van der Valk et al. [89] Pre-post study Netherlands	Community mental health services	Mindfulness Based Intervention, *n* = 19	None	2 × 60 min session/week for 4 weeks	Psychotic symptoms (SCL-90, PANSS)	4. Quantitative Descriptive	75
43. Yulina Astuti et al. [90] Non- Randomised controlled trial Indonesia	Inpatient	Mindfulness therapy, *n* = 27	TAU, *n* = 27	1 × 30 min session/week for 2 weeks	Hope (SHS-9)	3. Quantitative non-andomised	50

BCIS, Beck Cognitive Insight Scale [91]; BPRS, Brief Psychiatric Rating Scale [92]; BPRS-E, The Brief Psychiatric Rating Scale-Expanded [93]; CERQ, Cognitive Emotion Regulation Questionnaire [94]; CORE, Clinical Outcomes in Research Evaluation [95]; DASS-21, Depression Anxiety Stress Scale [96]; DERS, Difficulties with Emotional Regulation Scale [97]; HHS, Herth Hope Scale [98], Links Stigma Scale [99]; PANSS, The Positive and Negative Syndrome Scale [100]; SCL-90, The Symptoms Checklist 90 [101]; SCWT, Stroop Color Word Test [102]; SHS-9, Schizophrenia Hope Scale-9 [103]; WHOQOL-BREF, World Health Organisation Quality of Life-BREF [104]

#### Amount of formal home practice

Twenty-six out of the 43 studies (60%) described guidance given for home practice to participants within the treatment protocol (Table 2). Sixteen of these studies (16/26) included formal home practice as a core part of the intervention and set home practice assignments, with the remaining 10 studies encouraging home practice but not setting weekly assignments. Resources to help support participants to complete home practice included providing CDs of guided practises, written scripts, handouts, and forms to record home practice. Only five studies reported amount of home practice in a format that allowed for calculation of average numbers of minutes/day, or days/week [18–22] The frequency which people reported home practice ranged from 3 to 7 days/week, and the average duration of each home practice ranged from 1 to 30 min (we did not calculate a pooled estimate due to heterogeneity between studies, and the sparse data available). None of these five studies reported any analyses which examined the association between home practice completion and treatment outcome.

**Table 2:** Studies which included instructions/guidance on home practice (n = 26)

References	Guidance for home practice	Resources given for home practice	Is home practice measured (if so, how?)	Proportion of participants who report any formal home practice	Average reported formal home practice (Practice per day, or average number of practices per week)
1. Brown et al. [18]	Home practice core requirement	Guided meditation CDs	Yes (not stated how)	14/15 participants	24.71 min/day, *SD* 18.44, Range = 0.88–64.14
2. Çetin and Aylaz [60]	Home practice core requirement	Guided meditation CDs and booklet	No	Not recorded	N/A
3. Chadwick et al. [4]	Encouraged but not required	Guided meditation audiotapes	No	Not recorded	N/A
4. Chadwick et al. [61]	Encouraged but not required	Guided meditation CDs	No	Not recorded	N/A
5. Chadwick et al. [62]	Encouraged but not required	Guided meditation CDs	No	Not recorded	N/A
6. Chien and Lee [63]	Home practice core requirement	Not stated	No	Not recorded	N/A
7. (a) Chien and Thompson [64] (b) Wang et al. [65] (c) Chien et al. [66] (d) Chien et al. [67] (e) Chien et al. [68]	Home practice core requirement	Not stated	No	Not recorded	N/A
8. Davis et al. [19]	Encouraged but not required	Guided meditation CDs	Yes (self-report)	5/5 participants	2 ps reported doing daily one-minute breathing spaces 3 ps reported 5–30 min of daily practice
9. Davis et al. [20]	Home practice core requirement	Guided meditation CD and homework forms	Yes (record form handed in each week)	Not stated	28.72 min on an average of 54% of days
10. Jacobsen et al. [23]	Encouraged but not required	Guided meditation CDs	No	Not recorded	N/A
11. Johnson et al. [21]	Home practice core requirement	Guided meditation CDs	Yes (self-report)	Not recorded	Mean 3.7 days/week (*SD* = 1.4) 19.1 min per practice (*SD* = 14.6)
12. Jones et al. [73]	Home practice core requirement	Audio recordings	No	Not recorded	N/A
13. Lam et al. [22]	Home practice core requirement	Guided meditations on MP3 players, practice manual and logbook	Yes (record form handed in each week)	14/26 participants (according to ITT analysis)	Mean 31 min per week (SD = 17.34, range 0–86.4 min per week)
14. Langer et al. [74]	Home practice core requirement	Guided meditation CDs and homework forms	No	Not recorded	N/A
15. Lee [76]	Home practice core requirement	Not stated	No	Not recorded	N/A
16. López-Navarro et al. [77]	Encouraged but not required	Guided meditation audiotapes	No	Not recorded	N/A
17. (a) López-Navarro et al. [78] (b) López-Navarro et al. [79]	Encouraged but not required	Guided meditation audiotapes	No	Not recorded	N/A
18. MacDougall et al. [80]	Home practice core requirement	Not stated	No	Not recorded	N/A
19. Martin et al. [81]	Encouraged but not required	Mindfulness scripts and hand outs	No	Not recorded	N/A
20. Mediavilla et al. [82]	Home practice core requirement	Audio recordings	No	Not recorded	N/A
21. Özdemir and Budak [83]	Home practice core requirement	Booklet	No	Not recorded	N/A
22. Randal et al. [84]	Home practice core requirement	Guided meditation CDs and hand-outs	No	Not recorded	N/A
23. Ryan et al. [85]	Home practice core requirement	Wallet cards/worksheets for skills practice	No	Not recorded	N/A
24. Samson and Mallindine [25]	Encouraged but not required	Not stated	No	Not recorded	N/A
25. Sheng et al. [46]	Home practice core requirement	Not stated	Yes (record form handed in each week)	Not stated	N/A
26. Van der Valk et al. [89]	Encouraged but not required	Guided meditation CDs	No	Not recorded	N/A

### Secondary review question: what are the reported facilitators/barriers to formal home practice for people taking in a mindfulness for psychosis group? (Qualitative)

#### Study characteristics

We extracted qualitative data where available from the 11/43 studies included in the primary research question, categorised as either fully qualitative or mixed methods [18–21, 23–29]. Additional searches for solely qualitative studies resulted in an additional 6 relevant papers being identified (Additional file 1: Fig. S3). These additional qualitative papers, all conducted in the United Kingdom (UK), were conducted across a range of service settings, including an acute inpatient ward [30], an early intervention service [31], and community mental health services [32–35]. Three used grounded theory as the qualitative analytical approach [31, 34, 35], 2 used thematic analysis [30, 33], and 1 used interpretative phenomenological analysis (IPA) [32]. A total of 17 studies were therefore included in this qualitative part of the review.

#### Reported facilitators and barriers

We began the data synthesis by looking at the 10/17 included studies which were fully qualitative (as opposed to mixed-methods), on the basis that they were likely to yield the richest data. Our first observation was that these studies had primarily focused on participants’ experiences of the group itself, and in-session mindfulness practice, and most did not include any specific questions in their interview schedule about home practice. Perhaps unsurprisingly therefore, four studies did not report any qualitative data at all relating to home practice [31–34]. Brown et al. did include a specific question about “barriers” participants might have encountered in completing home practice in their study [18]. Participants mentioned difficulties in fitting their mindfulness practice into their daily schedule, and difficulties in their home environment which made it more difficult to practise e.g. noise and disturbance in shared accommodation. Similar themes were reported by McHale et al. in terms of successful completion of home practice being contingent on the ‘right’ conditions (e.g. it being easier when feeling relaxed, and when the home environment was quiet vs. feeling agitated, and/or there being noise around at home) [35]. Some participants in an inpatient study spontaneously reported practising between sessions, and intending to carry this on at home after discharge as it had been beneficial for them: “well it’s just got me through so much, you know? I’ll carry on with it even if it’s at home on my own”—([30], pg.606). This was also reported in another inpatient study, within the theme of ‘transfer to everyday life ([28], pg.10)’: “This exercise was much easier to implement [than Progressive Muscle Relaxation], and that's why I find it much better. I can do it at home alone.” This may have been an over-optimistic goal however, as participants in community groups often reported more ambivalence towards completing home practice, for example in saying they struggled to make regular time for it in their schedules [19].

We then moved on to reviewing the data extracted from the remaining mixed-methods studies, to build on our initial synthesis of findings. We found that the majority of mixed-methods studies yielded limited qualitative data from feedback forms or brief interviews, and did not report any findings specifically about home practice [20, 21, 23–26, 36–38]. The one exception to this was the study by Tong et al. [27], who conducted a full grounded theory analysis of the experience of 11 group participants. The paper reported 2 relevant themes on home practice; ‘Difficult to practice outside of group’ and ‘Stressful when discussing home practice’. The latter theme was illustrated by the following quote in the paper: “Perhaps I’m too lazy and didn’t spend much time at home practicing, so when I’m being asked about the homework, I felt some pressure.” (pg. 557). This is interesting as it highlights the potential for home practice to trigger feelings of shame or high levels of self-criticism when participants feel they are failing to complete it. This links back to Chadwick’s original stance on home practice in mindfulness for psychosis groups which was that too much emphasis on home practice could be counter-productive in this clinical population due to a greater sensitivity to perceived failure experiences [39].

In summary, we found that most mindfulness-based interventions for psychosis studies did mention home practice in their protocols, with over a third (37%) including home practice as a core requirement and setting regular home practice. However, very few studies measured or reported how much home practice people were doing. It is therefore not currently possible to say how much home practice people do when undertaking a mindfulness for psychosis group, or whether completion of home practice is linked to better treatment outcome. Qualitative data is also lacking on what people’s experiences of home practice are, and specifically what might help (facilitate) or hinder them (barriers) to complete home practice. The aim of study 2 was therefore to conduct a qualitative study to fill this gap in our current knowledge.

## Study 2: Qualitative study of facilitators and barriers to home practice

### Method

#### Research question

What are the self-reported facilitators and barriers to people doing home practice during a mindfulness-based interventions for psychosis group?

#### Participants and procedure

Participants were recruited from a Community Mental Health Team (CMHT) in a UK NHS Trust where 8-week mindfulness for psychosis groups were offered to service users as part of routine care (see Jacobsen et al. [40] for a more detailed description of the group intervention). Home practice was encouraged as part of the groups but was not a core requirement. We used purposive sampling [41] as we were interested in selecting participants specifically for the particular characteristic that they had taken part in a mindfulness group at the CMHT. As information on home practice completion was not routinely recorded by the group therapists, it was not possible to sample purposively on the basis of whether people had or had not completed home practice. However, we made it clear that we were interested in talking to people who may or may not have done any home practice during their mindfulness group in order to learn more about a range of experiences. Eligible participants were approached to take part by a member of CMHT staff. Interviews were conducted over the phone, or in person at the CMHT team base, by a graduate research student or assistant psychologist, using a semi-structured interview schedule (see Additional file 1). The assistant psychologist (CM) worked as part of the clinical team at the CMHT, but was not previously known to any of the service users they interviewed, and also had not been involved with running the mindfulness for psychosis groups. The graduate research students (TC & KS) were not previously known to any of the participants and had never been part of the clinical team at the CMHT. Interviews were audio recorded for transcription, and the interviewers also took contemporaneous paper notes. Two peer experts, with lived experience of mental health difficulties, were involved in developing and reviewing all study materials, including the information sheet, consent form and interview schedule. This was to ensure all information was explained clearly to participants, and interviews were conducted with sensitivity and respect. For example, peer experts emphasised the importance of wording the interview questions sensitively so that participants did not feel they were being judged badly for not completing home practice, or finding it difficult. The study protocol was written and pre-registered on the Open Science Framework before recruitment started (https://doi.org/10.17605/osf.io/cgakp; 22nd May, 2019).

#### Data analysis

Data collection and analysis were conducted in parallel. Interviews were transcribed verbatim using Jeffersonian Transcription Notation [42], which includes annotations on transcripts to give extra information about conversational context, such as pauses in speech, or rising/falling pitch or intonation (see Additional file 1 for notation key). The data were analysed using thematic analysis to identify recurring patterns of meaning within the data (themes) and the relationship between the themes [43]. We adopted a critical realist stance, based on the principle that there is no single absolute reality and knowledge is subjective and mediated by individual perceptions and beliefs. We therefore selected thematic analysis as the most appropriate method of analysis as it is theoretically and epistemologically flexible [44]. In line with the study aims, and to stay close to participant’s narrative, the analysis derived themes predominantly on a descriptive and semantic level. Initial codes were generated, and then refined using an iterative process through re-reading transcripts and the subset of codes were sorted into themes and sub-themes. Each theme was then reviewed at the coding level by re-reading the data extracts and ensuring they fit into each theme and reviewed at the level of the theme to ensure each theme coherently links to other themes. During analysis, recordings were replayed and transcripts were re-read repeatedly to ensure the data supported the analysis.

#### Research team and reflexivity

The qualitative analysis was conducted by TC, KS, and PJ, none of whom were previously known to the research participants in any capacity, and had not been involved with running the mindfulness for psychosis groups at the CMHT. The research students (TC & KS) were undertaking a masters-level degree in applied clinical psychology, and had previous work experience in mental health services. Both had some limited personal experience of experiential mindfulness practice, including using a mindfulness app. To enhance transparency, they both kept reflexive journals when conducting the interviews, to reflect on how their multiple identities, life experiences, and beliefs and assumptions may be impacting on their interpretation of the emerging data. They were trained and supervised by PJ, a Clinical Psychologist and Mindfulness teacher, whose area of clinical and research expertise was psychosis.

### Results

#### Participant characteristics

Five people took part in the study. The sample comprised of 4 women and 1 man, all of whom had a diagnosis of a schizophrenia-Spectrum disorder (ICD-10 [45]; F20-F29), with an average age of 45 years old (range 24–60). All had taken part in a mindfulness for psychosis group at the CMHT within the last 12 months. We asked each participant to self-report how much home-practice they had completed during their mindfulness group, and since finishing the groups to provide additional contextual data for their interviews (Table 3). All participants reported completing at least some formal home practice during their group, and 4/5 reported still practising at least once a week.

**Table 3:** Participant details on frequency of mindfulness practice

Participant number	How long ago taken part in mindfulness group	On an average week, any formal home-practice done whilst taking part in mindfulness group (self-report)	On an average week, any formal home-practice done since finishing the mindfulness group (self-report)
1	Less than three months	At least once a week	At least once a week
2	Less than six months	At least every day	Never
3	Less than 12 months	Several times a week	At least once a week
4	Less than 12 months	Several times a week	Several times a week
5	Less than one month	At least every day	At least every day

#### Qualitative findings

**Fig. 2:**
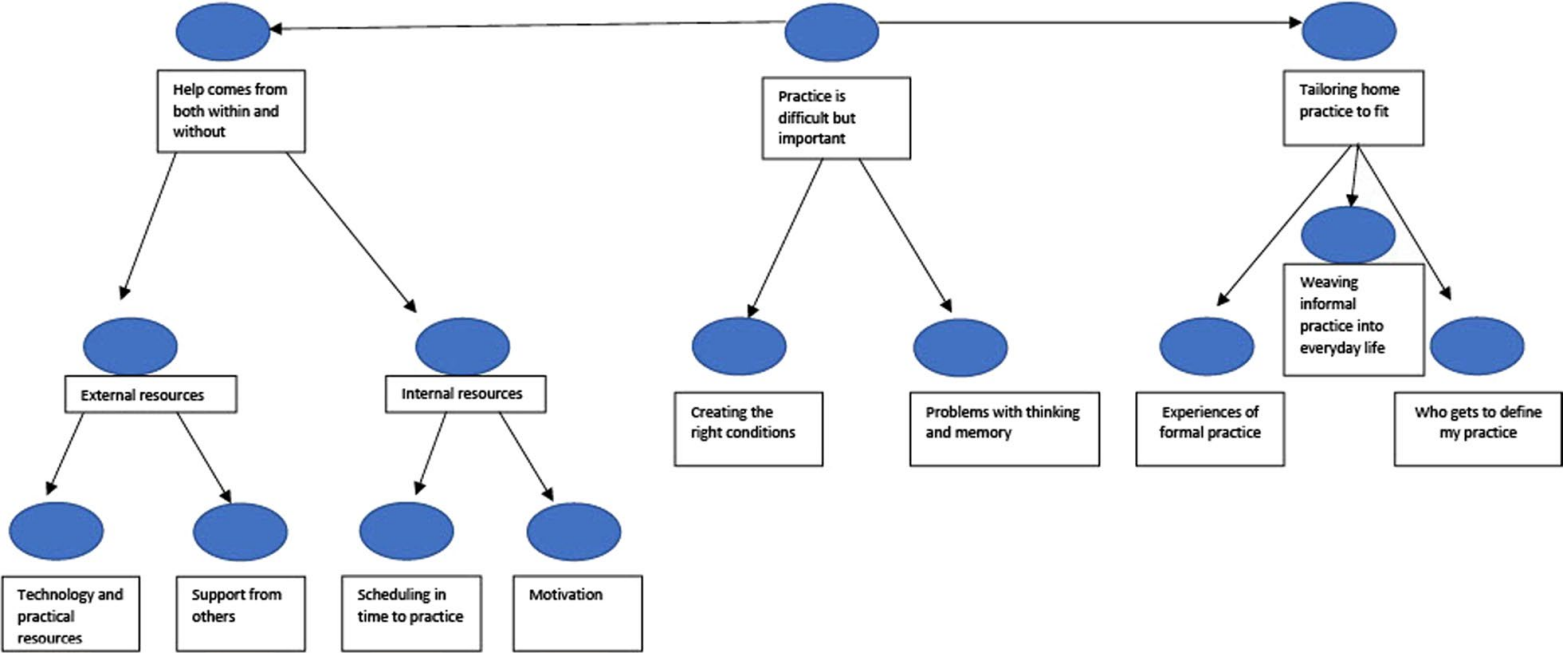
Qualitative results thematic map: the territory of home practice

We entitled our thematic map ‘the territory of home practice’ as this encapsulated our exploration with the participants in ‘mapping out’ their experiences (Fig. 2). Our final model had three main themes; (1) *Practice is difficult but important* (central theme), (2) *Tailoring home practice to fit*, and (3) *Help comes from both within and without.* Central findings are summarised below.

1. Practice is difficult but important

Participants viewed home practice as important, and a key part of them benefitting from the mindfulness group in the longer term. However, participants also experienced conflict in knowing home practice would be helpful, but finding it hard to do for various reasons. A sub-theme related to ‘creating the right conditions’. The ‘right’ conditions related to both the external ‘weather pattern’ (e.g. noise coming from the street or children at home being distracting), and the internal weather, such as the presence of physical pain, and difficult thoughts and emotions.

if I’m trying to be mindful (.) sometimes (.) if I’m in a bad mood (.) it makes me a lot more aware of troubling thoughts (.) and you know (.) that I sometimes might not acknowledge (.) you know (5) so although I do still use mindfulness (.) you know (.) sometimes it’s not always a peaceful experience (3) **(P3)**

There were few direct references to psychotic symptoms specifically, although some participants did mention the presence of voices during practice, or thoughts about being unsafe.

and (.) also (.) things like (.) if I heard a helicopter overhead (.) cause that’s one of things (.) you know (.) I fear they were monitoring me (.) or something **(P3)**

However, there was no special ‘positioning’ of psychotic experiences; for example, one participant said that they were just as likely to be put off from practising when their pain from a chronic health condition was bad, as they would from how their mental state was. One participant also reflected on how mindfulness practice was anathema to the ‘cult of busyness’, in that it could seem wasteful or indulgent to sit and deliberately cultivate ‘non-doing’.

□Exactly (2) yeah because (.) you know (.) with mindfulness (.) the biggest block is that you can’t see the effect (.) they’re VERY subtle (2) so even after you finish mindfulness session (.) you’re like (.) why did I just waste my time ((laughs)) **(P4)**

Problems with memory or thinking were mentioned by three out of five participants e.g. “cause I forget a lot (3) so maybe forgetfulness (.) always forgetting things” **(P1).** These difficulties were not ascribed to any particular cause, but were viewed as fairly long-standing difficulties, which participants were used to having to work around and adapt to.

2. Tailoring home practice to fit

An unexpected finding which arose in the analysis was that participants often challenged the conventional definition of ‘formal’ and ‘informal’ practice which we set out for them at the beginning of each interview script ‘(sub-theme of ‘who gets to define my practice’). For example, P1 described a ‘soles of the feet’ practice on the bus on her way home from a mindfulness group session, and how they viewed this as ‘formal’ rather than informal, given their intention to turn towards their experience in a mindful attitude. Participants also described using practises in a way which was helpful to them, even if they were aware this not necessarily the intended purpose. For example, falling asleep is a common experience in mindfulness practises (particularly when lying down, such as in the body scan). One participant described using this to their advantage, even though they were aware this not the intended purpose necessarily.

I- I-I I wouldn’t say it’s mindfulness (.) cause my intention (.) would be to- to- ur:::m (2) make me more relaxed (.) where I-I-I sleep better (.) it’s not- it’s not like a (2) I don’t do it intentionally (.) to- urm- to- as a- (.) I don’t do it as an (.) intentional practice (2) it’s more of a (.) just a tool for me to go to sleep **(P4)**

The sub-theme of ‘weaving informal practice into everyday life’ related to how people applied the mindfulness skills, and attitudinal qualities (e.g. curiosity, compassion) into everyday experiences and how beneficial they had found this. The descriptions of noticing the mundane and everyday were frequently very joyful and light-hearted as P2 describes here.

well I do the informal one (.) you know (.) I do- I do- these with my kids (2) and I tell them (.) □OH (.) the sun is SHINING (2) the birds are singing (.) it’s a beautiful day (.) make every second count **(P2)**

3. Help comes from both within and without.

Participants valued both internal resources e.g. their own motivation, and their own strategies for completing home practice, as well as external resources, such as use of CDs and apps, and support from others. At the heart of home practice, was the belief that it was helpful, and the motivation to try to overcome difficulties which had led them to try the group in the first place.

need to get better (2) because I really wanted to (.) get out of (.) depression (2) and it- it did help (3) **(P2)**

Yeah (.) it’s more for my brain (.) really (.) that’s the best motivating thing (2) because I know it’s benefitting my brain (.) it’s like (.) when you’re eating healthy food and you feel good about yourself (.) so when I do it (.) I’m like giving rest to my brain (2) so that makes me feel good **(P4)**

Support from others, including family members, and the mindfulness group therapists, were important in supporting people to maintain their practice. The opportunity to attend monthly follow-up sessions was particularly valued as it helped provide an anchor to the group which stopped it being forgotten.

YEAH (.) that one I- I- I’m thinking (.) the group here actually reminds us to keep doing mindfulness (2) because (.) you know why we- we- (.) my memory is not very good (.) and if I stopped it (.) I’ll forget straight away (2) but when I come here (.) it reminds me **(P5)**

Support from other group members, both during the group, and in monthly follow-ups were particularly valued, with participants describing in very warm terms how they felt understood and inspired by others in the group with similar struggles.

□OF COU:::RSE that gives you more □wings to fly (2) you know (.) when you- when you notice (.) someone is pushing you to succeed (.) you know (.) someone looks (3) ur::m (.) cares about your wellness and everything (.) you know (.) it’s wings for you to fly ((laughs)) **(P2)**

## Discussion

This study aimed to review the current literature on home practice in mindfulness-based interventions for psychosis groups, and to investigate participants’ perspectives on the barriers and facilitators to home practice. We found that although over a third of studies in our review recommended home practice as either an optional or core requirement of the group (37%; 16/43), only five studies reported any data on how much home practice people were doing. The findings from our qualitative study suggested that home practice was challenging but valued.

Our findings that home practice is rarely reported in mindfulness for psychosis studies was surprising given that many did mention home practice as part of the group protocol, and supporting materials such as CDs and handouts, were commonly provided. In addition to the five studies which reported data on home practice [18–22], one additional study stated that home practice monitoring was conducted by way of weekly record sheets, but this data was not reported in the paper [46]. Monitoring or recording of home practice was not explicitly mentioned in the remaining studies. This does not necessarily mean that no data on home practice was collected; only that none was reported in the paper. There may have been a range of ways in which home practice was reviewed and used clinically within the groups, for example by participants giving verbal reports when reviewing home practice in the group, without formal records being kept by participants or teachers.

Several factors may explain why homework was encouraged, and perhaps even discussed and recorded (but not reported) in these studies. Clinicians and researchers may believe that home practice is not likely to be associated with better treatment outcome in people with psychosis, hence there is no need to report data on it. However, this attitude would be surprising given previous meta-analyses including both clinical and non-clinical samples which suggest a clear link between increased homework completion and better therapeutic outcomes [11, 12]. An alternative explanation could be that home practice is considered potentially valuable, but concerns remain about the burden on participants in being asked to complete homework diaries or similar in a formal way.

From the available data, it is currently unclear to what degree a review of home practice was a standard part of the group curriculum (whether or not they reported data in the journal paper). Where home practice was not reviewed or monitored within the groups, this could be due to therapists’ concerns about potentially triggering shame or guilt in a vulnerable clinical population if they struggle to complete home practice, or to record it accurately. However, it is interesting to contrast this with the approach taken in Mindfulness based Cognitive Therapy (MBCT) for depression trials which have taken a more robust approach to recording homework e.g. [47]. Home practice is set and recorded each week, despite the fact that sensitivity to perceived failure and tendency towards self-criticism is commonly found in people with a history of depression [48, 49]. In fact, working with self-critical thoughts triggered by a ‘failure’ experience around home practice can offer an opportunity to practice compassionate responding in a mindfulness class, if handled skilfully by the group therapist. Participants in one of the earliest published mindfulness-based interventions for psychosis studies rated Yalom’s [50] therapeutic factor of ‘universality’ as one of the most important group factors which promoted a good therapeutic outcome [4]. Therefore, given how common struggles with home practice are, hearing that other group members are having similar challenges could be very normalising for people if home practice review is included in the weekly group curriculum.

The findings of the qualitative study in terms of barriers and facilitators to home practice are largely consistent with previous qualitative studies. We did not find any sense of special ‘positioning’ of psychotic symptoms, such as voices or paranoid thoughts, although some mention was made of these experiences in the interviews (e.g. the sound of a helicopter overhead triggering worries about being monitored). Participants mostly described ‘everyday’ barriers to home practice, such as children being at home, or noise coming from the street. This fits with previous qualitative studies in psychosis which mention noisy home environments, or external interruptions, as barriers to home practice [18, 35]. Furthermore, our participants reflected on the role of the ‘internal’ environment, given how mindfulness involves turning towards what is present; therefore it is challenging to practice when this involves coming into contact with unwanted emotional states, although arguably this is when mindfulness skills can be honed most effectively. This also fits with the findings of the general community study in which people described a reluctance to practice when they felt bored or irritated because these are difficult emotions to sit with [13]. In terms of facilitators to practice, a belief that mindfulness was helpful, and a motivation to ‘get better’ helped people to at least try to practice at home, or when out and about. Practical resources such as the audio recordings provided, and also support from family, and from the mindfulness group were also valued.

These findings are consistent with the COM-B model [51], in which behavioural change is theorised to arise from people’s capability, motivation and opportunity to successfully implement the change. In mindfulness for psychosis groups, participants are perhaps viewed as *motivated* to practice at home and are given the *opportunity* to do so (i.e. through setting homework practices and providing guided meditations on CDs/digital files). However, people with psychosis may not have hitherto been seen as *capable* of home practice, hence the reluctance to monitor or record home practice effectively. This may arise from benevolent paternalism [52], which although well-intentioned, may actually harm patients by limiting the effectiveness of therapies they receive by blocking access to certain components which are deemed ‘risky’, such as home practice.

With regards to clinical implications, Masheder et al. [53] further use the COM-B model as a framework for seven proposed factors that mindfulness teachers can use to help them support participants in completing home practice: (1) self-efficacy; (2) self-care; (3) beliefs about practice; (4) planning/commitment; (5) social support; (6) the relationship with the teacher and; (7) experiencing the rewards of practice. This framework can be a useful guide to ensure effective implementation of behavioural change through addressing each factor, such as encouraging participants to plan ahead to make time to practice, and framing practice as an act of self-care, rather than a chore. Our findings would suggest these factors should apply in mindfulness for psychosis groups, as in other mindfulness groups, and their effectiveness should be further explored in future studies.

In terms of strengths and limitations of the current study, we pre-specified and published our search strategy and review protocol in advance, and defined ‘mindfulness-based interventions for psychosis’ groups quite broadly for the sake of maximising the breadth of the systematic review. We did not search the grey literature however, or unpublished theses, so it is possible there were relevant studies outside the peer-reviewed literature which were not included in our review.

For the qualitative study, we purposively sampled people who had taken part in a mindfulness for psychosis group, but we were not able to specifically select people depending on whether they had or had not completed any home practice. Although we endeavoured to convey our interest and enthusiasm in speaking to people who had both done, and not done, any home practice, it is possible that people who had found home practice extremely difficult would not have felt comfortable volunteering to participate in the study. Further work would therefore need to be done in proactively seeking to recruit such participants in qualitative studies, to understand the experiences of people who do not do any home practice at all, and the reasons underlying this. We sampled people all from the same service (although not all from the same group cohort), which allowed us to explore experiences in one particular context in some depth, as is the aim of qualitative research, rather than to ‘generalise’ as in the positivist framework more commonly applied in quantitative research [54]. We also do not make any claims in relation to have achieved ‘data saturation’, as this is a inconsistently defined term in qualitative research [55, 56], and sample size may be better informed by ‘information power’ [57], which focuses on key dimensions such as study aim and sample specificity. Our study aim was relatively narrow, and the participants held highly specific characteristics in relation to the study aim (i.e. people who had recently completed a mindfulness for psychosis group), hence we regarded each ‘case’ as having high information power [58].

## Conclusions

We highly recommend future mindfulness for psychosis group studies record and report home practice more thoroughly, so that the link between home practice and treatment outcome can be effectively evaluated, and treatment guidelines can be updated accordingly.

## Supplementary Information

**Additional file 1.** 1. Search Terms. 2. Figure S3. PRISMA diagram for secondary searches (qualitative). 3. Semi-structured interview topic guide. 4. Key to Jeffersonian Transcription Notation.

## Data Availability

The datasets used and/or analysed during the current study are available from the corresponding author on reasonable request.

## Abbreviations

CDs: Compact discs
CMHT: Community Mental Health Team
COM-B model: Capability, opportunity, motivation, and behaviour model
ICD-10: International Classification of Diseases-10th edition
IPA: Interpretative phenomenological analysis
MBSR: Mindfulness-Based Stress Reduction
MBCT: Mindfulness-Based Cognitive Therapy
MMAT: Mixed Methods Appraisal Tool
NHS: National Health Service
PRISMA: Preferred Reporting Items for Systematic Reviews and Meta-Analyses
RCTs: Randomised controlled trials
TAU: Treatment as Usual
UK: United Kingdom
