# Supplementary material for: Home practice in mindfulness-based interventions for psychosis groups: a systematic review and qualitative study
Source: BMC Psychol. 2022 Jan 12;10:9. doi: 10.1186/s40359-021-00694-4 (PMC8756717; doi:10.1186/s40359-021-00694-4)
Supplement: Supplementary file 1 — Additional file 1. 1. Search Terms. 2. Figure S3. PRISMA diagram for secondary searches (qualitative). 3. Semi-structured interview topic guide. 4. Key to Jeffersonian Transcription Notation. [file 40359_2021_694_MOESM1_ESM.pdf]

Title: Home practice in Mindfulness for Psychosis groups: A Systematic Review and Qualitative Study

Journal: BMC Psychology

Authors: Pamela Jacobsen[1*], Twinkle Choksi[1], Katherine Sawyer[1], Cassia Maximen[2], Emma Harding[2], Matthew Richardson[2]

[1] Department of Psychology, University of Bath, Bath, UK

[2] South London and Maudsley NHS Foundation Trust, London, UK

Corresponding Author: Dr. Pamela Jacobsen, Department of Psychology, University of Bath, Bath, UK, BA2 7AY (pcj25@bath.ac.uk), Tel: +44 1225 385091

Additional File 1

1. Search Terms
2. Figure 3 – PRISMA diagram for secondary searches (qualitative)
3. Semi-structured interview topic guide
4. Key to Jeffersonian Transcription Notation
5. PRISMA Checklist

Search terms

<u>Primary search terms – Mindfulness for Psychosis</u>
<u>PubMed:</u> (("mindfulness"[MeSH Terms] OR "mindfulness"[All Fields]) OR ("meditation"[MeSH Terms] OR "meditation"[All Fields])) AND (("schizophrenia"[MeSH Terms] OR "schizophrenia"[All Fields]) OR psychotic[All Fields] OR ("psychotic disorders"[MeSH Terms] OR ("psychotic"[All Fields] AND "disorders"[All Fields]) OR "psychotic disorders"[All Fields] OR "psychosis"[All Fields]) OR ("paranoid disorders"[MeSH Terms] OR ("paranoid"[All Fields] AND "disorders"[All Fields]) OR "paranoid disorders"[All Fields] OR "paranoia"[All Fields]) OR ("delusions"[MeSH Terms] OR "delusions"[All Fields] OR "delusion"[All Fields]) OR ("hallucinations"[MeSH Terms] OR "hallucinations"[All Fields] OR "hallucination"[All Fields]) OR "voice hearing"[All Fields] OR "distressing voices"[All Fields])

<u>Scopus:</u> (TITLE-ABS-KEY (*mindfulness*) OR TITLE-ABS-KEY (*meditation*) AND TITLE-ABS-KEY (*schizophrenia*) OR TITLE-ABS-KEY (*psychotic*) OR TITLE-ABS-KEY (*psychosis*) OR TITLE-ABS-KEY (*paranoia*) OR TITLE-ABS-KEY (*delusion*) OR TITLE-ABS-KEY (*hallucination*) OR TITLE-ABS-KEY (*"voice hearing"*) OR TITLE-ABS-KEY (*"distressing voices"*))

<u>Secondary search terms – Mindfulness for Psychosis</u>
<u>PubMed:</u> ((("mindfulness"[MeSH Terms] OR "mindfulness"[All Fields]) OR ("meditation"[MeSH Terms] OR "meditation"[All Fields])) AND (("schizophrenia"[MeSH Terms] OR "schizophrenia"[All Fields]) OR psychotic[All Fields] OR ("psychotic disorders"[MeSH Terms] OR ("psychotic"[All Fields] AND "disorders"[All Fields]) OR "psychotic disorders"[All Fields] OR "psychosis"[All Fields]) OR ("paranoid disorders"[MeSH Terms] OR ("paranoid"[All Fields] AND "disorders"[All Fields]) OR "paranoid disorders"[All Fields] OR "paranoia"[All Fields]) OR ("delusions"[MeSH Terms] OR "delusions"[All Fields] OR "delusion"[All Fields]) OR ("hallucinations"[MeSH Terms] OR "hallucinations"[All Fields] OR "hallucination"[All Fields]) OR "voice hearing"[All Fields] OR "distressing voices"[All Fields])) AND (qualitative[All Fields] OR experience[All Fields] OR ("feedback"[MeSH Terms] OR "feedback"[All Fields]) OR mixed-methods[All Fields] OR ("interview"[Publication Type] OR "interviews as topic"[MeSH Terms] OR "interview"[All Fields]))

<u>Scopus:</u> (TITLE-ABS-KEY (*mindfulness*) OR TITLE-ABS-KEY (*meditation*) AND TITLE-ABS-KEY (*schizophrenia*) OR TITLE-ABS-KEY (*psychotic*) OR TITLE-ABS-KEY (*psychosis*) OR TITLE-ABS-KEY (*paranoia*) OR TITLE-ABS-KEY (*delusion*) OR TITLE-ABS-KEY (*hallucination*) OR TITLE-ABS-KEY (*"Voice hearing"*) OR TITLE-ABS-KEY (*"distressing voices"*) AND TITLE-ABS-KEY (*qualitative*) OR TITLE-ABS-KEY (*experience*) OR TITLE-ABS-KEY (*feedback*) OR TITLE-ABS-KEY (*mixed-methods*) OR TITLE-ABS-KEY (*interview*))

Figure 3. PRISMA flow chart for additional searchers conducted to address secondary review question

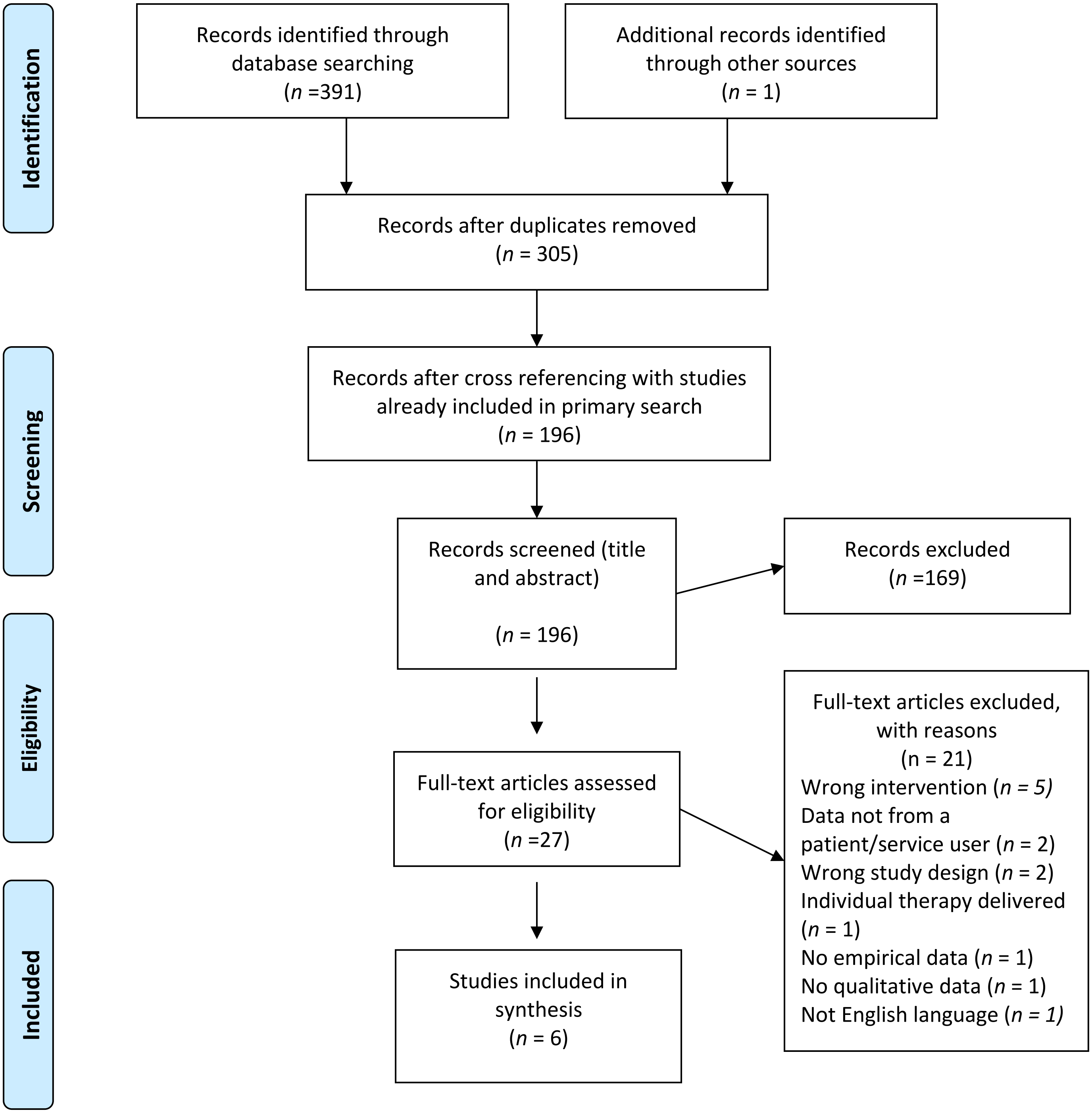

<u>**Home practice during a mindfulness group**</u>

Semi-structured interview topic guide
(prompts listed below the question)

Introduction

I am going to ask you a few general questions about whether you did any mindfulness practice in between the group sessions you took part in, and what might have helped with this, or got in the way. It is important to remember there are no right or wrong answers. I am really interested in your experience and your opinions, whatever they may be.

We are interested in different types of mindfulness practice people might do in between sessions. This might include what is sometimes called 'formal' practice, which means setting aside a set amount of time to intentionally practice mindfulness, such as sitting down at home to do a 5-minute mindfulness of the breathing practice. This is like the mindfulness exercises you would have done in the mindfulness group. There is also something which is called 'informal' practice, which is something you might do during your daily life or routine. This might include for example, noticing the taste of your toothpaste when you brush your teeth in the morning, or noticing bird songs as you walk along the street.

1) Did you ever do any kind of mindfulness practice in between the group sessions?
- Recap what formal and informal practice is
- If no, confirm this is for formal and informal practice and ask question 2a
- If yes, ask question 1a

1a) ***On an average week, did you do any formal practice?***
Recap what formal practice is
If no, rate as 1. If yes, on an average week, how often was this?

1	2	3	4
Never	At least once a week	Several times a week	At least every day

Prompts/probes:
- Explore any examples of formal and/or informal practice

2a) ***If little or no home practice done between sessions*:**
Just as a reminder, we are not judging people in any way whether or not they managed to do any home practice. We are just interested in learning more about different people's experiences. So for you, can you tell me a bit more about why you didn't do any/much practice in between sessions? Did anything get in the way for you?

2b) ***If some or a lot of home practice done between sessions*****:**

Was there any particular reason you did do some practice in between sessions?
Did anything make it easier for you?

3) Was there anything that might have made it easier for you to do some practice at home in between sessions, or that would have made it easier for you to practice if you had wanted to?

4) Finally, we are also interested in finding out about whether people have continued practicing mindfulness after the end of the group sessions. Have you continued doing any formal or informal mindfulness practices?

- Recap what formal and informal practice is
- If no, confirm this is for formal and informal practice and ask prompts and then move onto question 5
- If yes, ask question 4a

4a) ***On an average week, how often do you do any formal practice since finishing the group?***

Recap what formal practice is
If no, rate as 1. If yes, on an average week, how often is this?

1	2	3	4
Never	At least once a week	Several times a week	At least every day

Prompts/probes:
- What has helped?
- What has got in the way?
- What has stopped you from doing this?

Reasons might include:
- Support from peers/family/friends
- Use of audio recordings from groups
- Using online resources or mindfulness applications

5) That was all the questions I had for you today. Was there anything else you thought was important to tell me about your mindfulness practice which we have not already covered?

Key of Jeffersonian Transcription Notation

[...] = Brackets with ellipsis = Two related data extracts spoken close to one another

(text) = Single parentheses = What the individual is referring to, directly stated close to the data extract

(# of seconds) = Time pauses = A number in parentheses indicates the time, in seconds, of a pause in speech

(.) = Micropause = A brief pause, usually less than 0.2 seconds

↓ = Down arrow = Indicates falling pitch

↑ = Up arrow = Indicates rising pitch

- = Hyphen = Indicates an abrupt silence or interruption in utterance

° = Degree symbol = Indicates whisper or reduced volume speech

ALL CAPS = Capitalised text = Indicates shouted or increased volume speech

_ = Underlined text = Indicates the speaker is emphasising or stressing the speech

::: = Colon(s) = Indicates prolongation of an utterance

((text)) = Double parentheses = Annotation of non-verbal activity

[examples removed due to the possible identification of participants true identity] = Brackets with specific text = Included for ethical purposes

PRISMA 2009 Checklist

Section/topic	#	Checklist item	Reported on page #
**TITLE**			
Title	1	Identify the report as a systematic review, meta-analysis, or both.	1, 2
**ABSTRACT**			
Structured summary	2	Provide a structured summary including, as applicable: background; objectives; data sources; study eligibility criteria, participants, and interventions; study appraisal and synthesis methods; results; limitations; conclusions and implications of key findings; systematic review registration number.	2
**INTRODUCTION**			
Rationale	3	Describe the rationale for the review in the context of what is already known.	4-5
Objectives	4	Provide an explicit statement of questions being addressed with reference to participants, interventions, comparisons, outcomes, and study design (PICOS).	6-7
**METHODS**			
Protocol and registration	5	Indicate if a review protocol exists, if and where it can be accessed (e.g., Web address), and, if available, provide registration information including registration number.	6
Eligibility criteria	6	Specify study characteristics (e.g., PICOS, length of follow-up) and report characteristics (e.g., years considered, language, publication status) used as criteria for eligibility, giving rationale.	6-7
Information sources	7	Describe all information sources (e.g., databases with dates of coverage, contact with study authors to identify additional studies) in the search and date last searched.	6
Search	8	Present full electronic search strategy for at least one database, including any limits used, such that it could be repeated.	Additional File 1
Study selection	9	State the process for selecting studies (i.e., screening, eligibility, included in systematic review, and, if applicable, included in the meta-analysis).	7
Data collection process	10	Describe method of data extraction from reports (e.g., piloted forms, independently, in duplicate) and any processes for obtaining and confirming data from investigators.	7
Data items	11	List and define all variables for which data were sought (e.g., PICOS, funding sources) and any assumptions and simplifications made.	7
Risk of bias in individual studies	12	Describe methods used for assessing risk of bias of individual studies (including specification of whether this was done at the study or outcome level), and how this information is to be used in any data synthesis.	7
Summary measures	13	State the principal summary measures (e.g., risk ratio, difference in means).	8
Synthesis of results	14	Describe the methods of handling data and combining results of studies, if done, including measures of consistency (e.g., I^2) for each meta-analysis.	8

PRISMA 2009 Checklist

Section/topic	#	Checklist item	Reported on page #
Risk of bias across studies	15	Specify any assessment of risk of bias that may affect the cumulative evidence (e.g., publication bias, selective reporting within studies).	7
Additional analyses	16	Describe methods of additional analyses (e.g., sensitivity or subgroup analyses, meta-regression), if done, indicating which were pre-specified.	N/A
**RESULTS**			
Study selection	17	Give numbers of studies screened, assessed for eligibility, and included in the review, with reasons for exclusions at each stage, ideally with a flow diagram.	8
Study characteristics	18	For each study, present characteristics for which data were extracted (e.g., study size, PICOS, follow-up period) and provide the citations.	Table 1
Risk of bias within studies	19	Present data on risk of bias of each study and, if available, any outcome level assessment (see item 12).	Table 1
Results of individual studies	20	For all outcomes considered (benefits or harms), present, for each study: (a) simple summary data for each intervention group (b) effect estimates and confidence intervals, ideally with a forest plot.	N/A
Synthesis of results	21	Present results of each meta-analysis done, including confidence intervals and measures of consistency.	N/A
Risk of bias across studies	22	Present results of any assessment of risk of bias across studies (see Item 15).	N/A
Additional analysis	23	Give results of additional analyses, if done (e.g., sensitivity or subgroup analyses, meta-regression [see Item 16]).	N/A
**DISCUSSION**			
Summary of evidence	24	Summarize the main findings including the strength of evidence for each main outcome; consider their relevance to key groups (e.g., healthcare providers, users, and policy makers).	11
Limitations	25	Discuss limitations at study and outcome level (e.g., risk of bias), and at review-level (e.g., incomplete retrieval of identified research, reporting bias).	21-22
Conclusions	26	Provide a general interpretation of the results in the context of other evidence, and implications for future research.	19-20
**FUNDING**			
Funding	27	Describe sources of funding for the systematic review and other support (e.g., supply of data); role of funders for the systematic review.	24

From: Moher D, Liberati A, Tetzlaff J, Altman DG, The PRISMA Group (2009). Preferred Reporting Items for Systematic Reviews and Meta-Analyses: The PRISMA Statement. PLoS Med 6(7): e1000097. doi:10.1371/journal.pmed1000097

For more information, visit: **www.prisma-statement.org**.
